# Massive Open Online Course Study Group: Interaction Patterns in Face-to-Face and Online (Facebook) Discussions

**DOI:** 10.3389/fpsyg.2021.670533

**Published:** 2022-03-02

**Authors:** Pin-Ju Chen, Yang-Hsueh Chen

**Affiliations:** ^1^Teacher Education Center, Ming Chuan University, Taoyuan, Taiwan; ^2^Institute of Teacher Education, National Chengchi University, Taipei, Taiwan

**Keywords:** study group, MOOCs, blended learning, Facebook, college students

## Abstract

Interaction has been regarded as a key design component in online and distance learning. In this study, we convened a student-led, blended mode (face-to-face and online/Facebook discussions) massive open online course (MOOC) study group to facilitate interactions for learning. Multiple data, including voice recordings, one-on-one interviews, video recordings, and artifacts were collected and analyzed to detect patterns of interaction in both face-to-face and online/Facebook settings, as well as student perceptions of the blended MOOC study group. Findings indicated that, overall, the blended mode MOOC study group was helpful for promoting communication, providing help, resolving problems, and exchanging ideas and information among group members. Moreover, face-to-face meetings and online discussions both might have exerted their unique strengths and functions in different learning situations for different learners. We recommend future studies continue to explore the tenability of the blended mode MOOC study group in different contexts, subject areas, and age groups, as well as examining group dynamics and interactions that transform MOOC learning into interactive, motivating, and fulfilling journeys among study group members.

## Introduction

In 2012, Coursera had only about 1 million registered users (Pappano, 2012); in 2020, the total enrollment has exceeded 70 million (Coursera, 2020). Nevertheless, massive open online courses (MOOCs) have yet to fulfill their promises to deliver high-quality education to the mass (Rossi and Gnawali, 2014; Chen and Chen, 2015). Rossi and Gnawali (2014) pointed out that the quality of MOOCs could be much enhanced by incorporating social interactions into their instructional design. This claim has been supported by the study of Hew et al. (2018) wherein student reflections in 18 highly rated MOOCs were analyzed, and interaction was found to be one of the most important design characteristics of MOOCs. Other studies (e.g., Hone and El Said, 2016; Gregori et al., 2018) also identified interaction as a key factor in learners’ completion of MOOCs and online courses in general.

Despite its importance, interaction remains limited in the design of MOOCs (Gamage et al., 2020). Margaryan et al. (2015) studied the interactivity of MOOCs by randomly sampling 50 xMOOCs and 26 cMOOCs for analysis. xMOOCs are usually content-based and linear online courses, whereas cMOOCs tend to be more decentralized and allow learners to explore the content or upload feedbacks more freely. They found that the elements of interaction design (i.e., collaborative learning, collective knowledge, and instructor feedback) were quite limited. For instance, only 2% of xMOOCs and about 26% of cMOOCs had collaborative activities in design. Moreover, only 10% of xMOOCs and less than half of cMOOCs asked learners to share knowledge with others. As with learner-instructor interaction, none of the instructors in those MOOCs gave feedback to students on specific activities or assignments. Analysis of discussion threads also indicated a paucity of in-depth feedback on learners’ performance.

Over the years, scholars have been exploring potentially effective tools and pedagogies to promote MOOC interactions, such as online forums (Wise, 2018), social media (Ostashewski et al., 2018; Anderson et al., 2020), and collaborative assignments (Verstegen, 2018). However, study group, particularly “study group in blended mode” (i.e., face-to-face + online) remains relatively under-researched in the MOOCs literature. In the following subsections, we will briefly introduce the concepts of the study group and blended learning in relation to MOOCs, followed by empirical studies that examine interaction patterns in the contexts of online learning and MOOCs.

### Study Group

The study group has long been suggested as a means to promote interactions and learning experiences (Zevenbergen, 2004; Chen and Chen, 2015). It can be defined as a small number of learners meeting together to work on problems for the purpose of learning (Zevenbergen, 2004). Cognitively, students may share strategies such as consulting experts or searching for multiple resources to complete their course assignments. Affectively, students can support and encourage each other which stimulates motivation and engagement. Van Der Karr (1994) argued that a study group facilitates collaborative learning *via* interactions among group members. This is particularly helpful for online learning since the lack of interaction between class members had been repeatedly reported as a common cause of online students’ isolation and burnout (Morgan and Tam, 1999).

In the MOOC context, Li et al. (2014) formulated 12 groups of 4–5 participants to watch course videos together. Those participants were generally satisfied with this learning approach. In addition, compared to learning by themselves, the participants maintained that learning with the group was more effective and motivating. Interestingly, the video watching styles (i.e., centralized video control and centralized display, distributed video control and centralized display, and distributed video control and distributed display) affected group interactions in terms of video-watching time, pause frequency, and the amount of speech. In another study, the Chen and Chen (2015) led a 6-week study group wherein four students met face-to-face weekly to share progress and discuss issues related to a MOOC they decided to learn together. The study group members were found to share learning strategies, broaden perspectives on the course content, and raise their cultural awareness. Furthermore, the group members increased their learning momentum and tendency to put the inner thoughts into real actions. They concluded that the MOOC study group could promote a sense of community in a group and the dynamics/effectiveness of the MOOC learning. More recently, Krasny et al. (2018) applied a study group in their MOOC design. Students, from all over the world, formulated 19 “local groups” to meet face-to-face locally and discuss the course materials, nine “bilingual groups” to meet face-to-face and/or online to overcome language barriers, and 13 “interest groups” to exchange ideas online for specific topics of interest. It was found that the study groups were helpful for students to overcome barriers of language, content, culture, technology, and time management, as well as developing strategies for cooperative learning. Notably, the social interaction that fostered cooperative and collaborative learning was identified as the key factor of the learning effects of the MOOC students. The above studies have provided support of the study group to enhance interaction and learning in the MOOC context; however, components or patterns of interaction in MOOC study groups remain under-researched and warrant further research.

### Blended Learning and Massive Open Online Courses

Blended learning can be referred to as a learning mode/model that mixes online learning and face-to-face meetings (Oliver and Trigwell, 2005). More specifically, in the blended learning context, students learn partly from the content delivered *via* the Internet, and partly from instructional activities offered at brick-and-mortar locations (Staker and Horn, 2012). While online courses have the advantages of mobility, fast sharing, and flexibility in course activities to cater to students’ learning needs and preferences, they have often been criticized for the lack of interaction, delayed feedback, and low completion rates (Garrison and Vaughan, 2008). On the contrary, a well-designed blended learning environment may enrich the learning materials, give access to knowledge easily and, maintain adequate social interactions and feedback at the same time (Osguthorpe and Graham, 2003). Stockwell et al. (2015) concluded that blended learning can keep the values of online learning while adding the benefits of face-to-face meetings (Alghamdi et al., 2019). Empirical studies (e.g., Cornelius et al., 2019) also found students’ motivation, engagement, and satisfaction to be higher in blended learning as compared to their on-campus counterparts.

Studies have been exploring mechanisms within blended learning to promote social interactions (McCarthy, 2010). For instance, Ebner et al. (2017) found that social interactions in face-to-face sessions promoted online interactions such as forum discussions. Students got familiar and made friends with each other in face-to-face meetings, and then carried their friendships and communications over to their online activities. Furthermore, social presence was found to be another important factor in online learning (Garrison et al., 2010). According to the Community of Inquiry model (COI), the educational experience was supported by the integration of social, cognitive, and teaching presence (Majeski et al., 2018). Amemado and Manca (2017) suggested that in online learning environments where teaching presence was limited, courses should be designed to increase the other two types of presentations to maximize the effectiveness of learning. For example, research showed that when designing appropriately, high order learning in the blended learning model could be facilitated along with the increase of cognitive presence (Akyol and Garrison, 2011). Similarly, the social presence in both virtual and face-to-face sessions aggregate together to leverage the overall social presence in blended learning. In turn, a social presence facilitates communications and a sense of community that fosters interactions and collaborations among students (Tu and McIsaac, 2002; So and Brush, 2008).

Given the benefits of blended learning to promote interactions and learning, “blended MOOCs (bMOOCs)” have arisen to integrate MOOCs with traditional university classrooms—despite that MOOCs are essentially designed as independent courses to be delivered online (Alghamdi et al., 2019). A commonly known example of this bMOOC approach is “flipped classroom,” where students watch videos and other content at home and practice working through them at school. Several bMOOC modes have been proposed to illustrate the typology of bMOOC (e.g., Montgomery et al., 2015; Brannan, 2016; Alghamdi et al., 2019; Defaweux et al., 2019). Most of these frameworks categorize bMOOCs by the percentage of virtual and face-to-face time in the course. For example, Brannan (2016) introduced a continuum of MOOCs in blended learning. One end of the continuum is “Guide on the Side” (i.e., student control of learning) that all course elements are delivered online and the instructors facilitate students’ learning by providing physical office hours. The other end is “Sage on the Stage” (i.e., teacher control) mode, where all course elements are delivered face-to-face in traditional classrooms, and some MOOC elements are used as supplementary content or activities. In the middle of the continuum is the “Shared Control” mode where MOOC content or activities are either supplementary or complementary. In this mode, instructors may hold face-to-face meetings and online sessions regularly. In another study, Defaweux et al. (2019) sorted three patterns of blended MOOCs, namely “Pendulum,” “Sandwich,” and “Tetris.” In “Pendulum” blended MOOCs, classroom meetings and MOOCs were held alternatively. For example, a classroom meeting followed by a MOOC session that followed another classroom meeting. In “Sandwich” blended MOOCs, sessions of one form of meetings were arranged between the sessions of the other form. For example, the course starts with face-to-face classroom meetings for 3 weeks, followed by 3 weeks of MOOC sessions, and ends with another 3 weeks of face-to-face classroom meetings. In “Tetris” blended MOOCs, a session of a MOOC course becomes a section of different courses. For example, the week 6–week 8 sessions of a MOOC can also be used in another course as the sessions for the first 3 weeks. In all these patterns, each time slot is either scheduled for online activities or classroom meetings, but not both.

The abovementioned “bMOOCs” are simply combing traditional classroom teaching with MOOCs as opposed to incorporating face-to-face and online instructions within a single MOOC. Indeed, with few exceptions such as the Krasny et al. (2018) study mentioned earlier, it is difficult to achieve blended learning in a single MOOC, as stated by Gynther (2016), “*Blended learning is possible only in concepts that are not massive, e.g., the so-called “Little Open Online Course” (LOOC), Small Private Online Course (SPOC)…, or in concepts combining a group of enrolled students on campus with global participants*” (p. 21). Despite this limitation, the concept of blended learning in MOOCs can be supported by locally formulated Meetup groups, or what we called MOOC study groups. We contend that the effects of these study groups could be further enhanced by the blending of face-to-face meetings and online/Facebook discussions to exert their full potential. Also, we believe that the “study group” approach would be even valuable in conditions that traditional classroom contexts are not available. Next, we will discuss interaction patterns in online learning and MOOCs, then we will outline the significance of the present study.

### Interaction Patterns in Online Learning

In the online learning literature, a strand of research focuses on students’ interaction patterns in order to examine the structure or levels of interaction that may shed light on the design and facilitation of online courses (Michinov and Michinov, 2008; Hou et al., 2009; Philip, 2010; O’Riordan et al., 2016). Hou et al. (2009) analyzed students’ online interaction patterns and found five categories of interaction, including (1) sharing/comparing information; (2) discovering and exploring dissonance or inconsistency among participants; (3) negotiating meanings/co-constructing knowledge (4) testing and modifying proposed synthesis or co-construction; and (5) agreement statement(s)/applying newly constructed meanings. Notably, 90% of the interactions in the online discussion contributed to students’ knowledge construction. In the MOOC environment, Wang et al. (2017) analyzed interaction patterns in a prominent cMOOC course. Using content analysis and their Connectivist Interaction and Engagement (CIE) Framework as a reference model, they found that student interactions could generally be mapped to the four levels of CIE, namely *operation*, *wayfindin*g, *sensemaking*, and *innovation*. Among the four levels, most interactions happened in wayfinding, and much fewer cases were found in the highest innovation level due to challenges of time and technology requirements. Interestingly, most wayfinding interactions were found on Twitter, while the majority of sensemaking and innovation interactions happened in blog postings. We speculated that such a difference in interaction patterns was influenced by the affordances of the communication tools that supported different types/levels of student interaction.

More recently, Tawfik et al. (2017) explored learner interaction patterns within a Chemistry MOOC on Coursera. Adopting Interaction Analysis Model (Gunawardena et al., 1997) for analysis, they found the interactions of the students (i.e., sharing and comparing information) remained at low levels in the studied MOOC. Moreover, the intensity of interaction was found to decrease over the 10 weeks of the course. They argued that the low interactions might be due to the high attrition rate and lacking high-level discourse activities. Based on these findings, they recommended a social dashboard (e.g., a webpage that provides information on post activity, popular post, peer contribution, etc.) and project-based group activities to promote social interactions among MOOC students.

### Gaps, Purposes, and Questions

Several problems/gaps have been identified after a review of the literature. First of all, although interaction has been identified as a key factor of online learning and MOOCs, it remains limited in a large portion of MOOCs. Studies are warranted to investigate feasible ways such as pedagogies or learning models to leverage interactions in MOOC learning. Secondly, while blended learning is more likely to achieve better learning outcomes than online learning counterparts, it is difficult to achieve in the MOOC learning context (Gynther, 2016). As mentioned earlier, the concept of blended learning in MOOCs (bMOOCs) can be supported by locally formulated MOOC study groups *via* blending face-to-face meetings and online discussions to promote interaction, social presence, and engagement. Thirdly, although the study group approach has long been widely implemented in education, it is generally overlooked in MOOC studies. What is more, while the limited studies (e.g., Chen and Chen, 2015; Krasny et al., 2018) have verified MOOC study groups as an effective approach to promote peer interaction and learning outcomes, the patterns of interaction within MOOC study groups remained unclear. Hou et al. (2009) argued that pattern-discovery research was important because it helped educators identify situations or challenges of students, based on which more adequate guidelines could be proposed to facilitate student interactions.

In the MOOC literature, Li et al. (2014) explored students’ video navigation patterns as an index of interaction; however, such “interaction” was broadly and quantitatively measured by time and frequency of operation (i.e., video-watching time, pause frequency, and the amount of speech) rather than detailed conversations, leader and member behaviors, and the discussion topics. We deem that, more detailed examinations with qualitative measures would provide even richer information about student interactions in MOOC study groups. Furthermore, examining interaction patterns in face-to-face and online settings, respectively, helps us gain a more nuanced understanding of (1) which kinds of interaction could be better supported in a single setting, and (2) how face-to-face and Facebook discussions can work together to meet students’ learning needs.

To address the aforementioned gaps, we intended to perform a more nuanced analysis of the patterns of study group interaction in face-to-face and online/Facebook settings. We applied qualitative approaches (e.g., interviews and video recordings) to document group members’ interactions and perceptions of face-to-face meetings and online/Facebook discussions. Three research questions were proposed to guide this study:

1. What is the pattern of MOOC study group interaction in face-to-face meetings?

2. What is the pattern of MOOC study group interaction in online/Facebook postings?

3. How do MOOC study group members perceive their experience of interactions in face-to-face and online/Facebook contexts?

It is worth noting that we adopted Facebook as the platform for online discussion. Facebook is one of the most commonly used social media around the globe. Research has shown that when properly used, Facebook can promote formal and informal learning among college students (e.g., Madge et al., 2009; Aydin, 2012; Kasket, 2012; Miller, 2013). Facebook has been found to be easy to use for sharing resources and ideas online (Wang et al., 2012), and it has become one of the most common ways to promote collaborative learning (de Villiers and Pretorius, 2013; Liu et al., 2016; de Lima and Zorrilla, 2017; Wang et al., 2017). Based on blended learning literature, we deem that face-to-face study group learners may benefit more by incorporating Facebook discussions to extend discussions and interactions. In other words, the same cohort can schedule face-to-face meetings and establish a virtual Facebook group to interact seamlessly without the constraint of time and space.

## Methodology

### Participants

This study adopted the qualitative case study approach (Stake, 1995; Yin, 2017) to gain perspectives of group interactions and learner perceptions within a MOOC learning context. We recruited participants from the audiences who participated in an open speech on campus about the current development of MOOCs. Those who were interested in hands-on experiences of MOOCs left their contact information to our research team. Later, we contacted the potential participants, explained the nature of this study, and invited them to join our MOOC study group. In the end, four college students, Omar, Burton, Elizabeth, and Maggie (all in pseudo names, see Table 1 for their demographic and ethnographic profiles) volunteered to participate. An initial interview indicated that the students who participated in this study wanted to gain real experiences on MOOCs instead of merely listening to the lecture. In addition, three out of the four members were in their senior year and they were about to graduate at the end of the semester. They had flexible schedules and wanted to make good use of their time to be better prepared for the future. The following are brief descriptions of the four participants based on the researchers’ observations:

**TABLE 1:** Participants’ demographic profiles.

	Omar	Burton	Elizabeth	Maggie
Gender	Male	Male	Female	Female
Year of college	Senior	Senior	Senior	Junior
Study major	Engineering	Material Science	Chinese Literature	Chinese Literature

### The Massive Open Online Course: An Introduction to Marketing

During our first group meeting, the participants were instructed to browse available courses on Coursera to determine a course to study together. The participants were encouraged to follow their passion and choose whichever they liked. They finally picked a 9-week course entitled “***An Introduction to Marketing***” offered by the Wharton School of the University of Pennsylvania and taught by professors Barbara Kahn, Peter Fader, and David Bell. When asked about their reasons to choose that course, the participants expressed that the course offered essential skills in the job market, which was especially helpful as three of the group members would graduate soon. A participant stated that she used to take Chinese literature courses in her own department; now she wanted to try something new. Although the MOOC had already started 2 weeks, our participants still had a chance to catch up because they had not missed any exam or assignment due dates.

According to the syllabus, the marketing course aimed to introduce the fundamental knowledge of marketing such as branding, advertising strategies, and new market entry. Major course components included video lectures, quizzes, and online discussions. The course was taught in English, and all the videos had English subtitles available. Each week, students were expected to watch six to eight lecture videos, answer small quiz questions embedded in the videos, and complete the assignments. It was estimated that 5–6 h per week were required to complete the study.

### Study Group Design and Facilitation

A total of 6 weekly meetings were scheduled on Thursday evenings at 6–8 p.m. (see Table 2 for more details). Based on our past experience, many course/platform functions would be ignored by students if the instructor failed to guide them carefully: some MOOC learners may never visit pages of the forum or even the course syllabus page. As such, in the first meeting, the researchers explained the concepts of MOOCs, helped the participants set up Coursera accounts, and walked them through the basic operations of the Coursera platform. As highlighted by Castaño-Muñoz et al. (2016), such an orientation session is crucial for equipping necessary skills and self-efficacy for subsequent online learning.

**TABLE 2:** Schedule of weekly group meetings.

Week	Main activities	Facilitator
1	Introduction to MOOC learning	Researchers
2	MOOC learner’s experience sharing; Group discussion	Researchers/invited speaker
3–6	Group discussion	Assigned member

In the second face-to-face meeting, a senior who had been actively participating in a previous MOOC study group was invited to share her own learning experience. Our intention was to help the group members set reasonable expectations and then determine the goals and rules/logistics of the study group on their own. Such self-regulated learning initiatives, particularly goal setting and planning of learning are critical for ensuring MOOC learning outcomes (Kizilcec et al., 2017).

The agenda of the subsequent 4 weeks was determined by the group members themselves, as our goals were to promote participants’ self-agency, as well as their commitment and responsibility. After the discussion, the group members decided to utilize the 2-h meeting time to discuss lessons and quizzes together. Upon the encouragement of the researchers, the participants also decided to take turns leading the weekly meeting, namely each person facilitated 1 week of discussion. In addition, the study group members created a Facebook group and in subsequent meetings, the researchers encouraged the participants to make good use of the Facebook group for asynchronous communications.

Usually, the first 2 weeks of the MOOC course are critical for building rapport and establishing rules (Tseng et al., 2016); therefore, the researchers facilitated the MOOC study group by modeling how to lead a meeting in the first 2 weeks. The researchers consulted scaffolding strategies suggested by previous research (e.g., Lim et al., 2014; Wang C. X. et al., 2014), such as inspiring active involvement and useful roles, encouraging group communications, summarizing and clarifying the content of the discussion, and acknowledging and connecting thoughts and feelings expressed.

In subsequent meetings (Weeks 3–6), the study group members took the responsibility to manage their discussions, including the agenda and ways of interaction. As mentioned earlier, the participants took turns leading the discussion; therefore, each of them had a chance to experience the roles of both participant and leader. During this student-led stage, the researchers attended the computer lab to introduce the meeting, but for the main meeting time, the researchers and two research assistants sat at the other side of the computer lab to observe student interactions. We did not interfere with group members’ discussions unless they came to ask questions or request assistance. After group members finished their meeting, the researchers spent 20–30 min leading a focus group discussion, in which we probed, summarized, and wrapped up the progress of the study group. The focus group guiding questions included, but were not limited to: “What is the focus of discussion or activities this week?” “What kinds of problems have you encountered and how did you resolve them?” “Which learning strategies have you discovered and shared?” and “What do you plan to do in the upcoming week(s)?”

In addition, the researchers took the following steps to promote communications in both online and face-to-face contexts. Firstly, we shared some personal feelings and experiences with the study group members, as past research found that facilitators’ disclosure of their personal lives could enhance the connections among group members and between members and the facilitator (Holt et al., 1998). In this study, the researchers shared some personal interests such as favorite music and technology gadgets for learning, and occasionally they launched or joined the participants’ informal chats about what happened at school or in society. Secondly, we facilitated connections between Facebook and face-to-face activities. For example, we took the chance to talk about what happened online when we met in person, and we also encouraged the participants to extend their discussions on Facebook by sharing information and feelings over there. Such endeavors may increase the social presence of the study group members, meanwhile promoting student learning through both online and offline interactions.

### Data Collection

**FIGURE 1:**
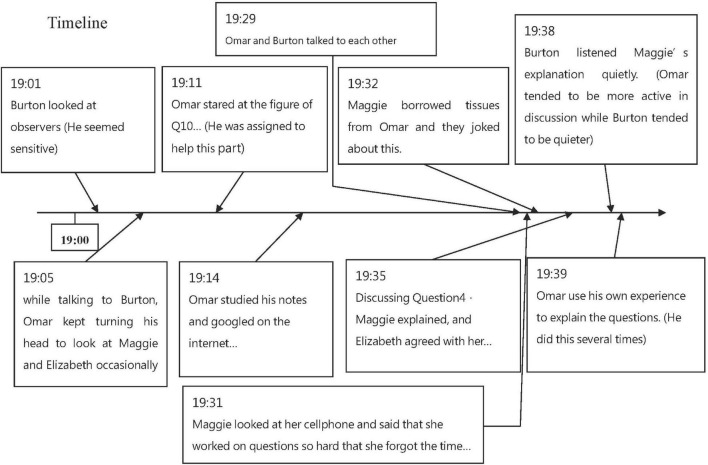
A snapshot of an observation note (week 4).

Data were collected *via* multiple sources, including observation notes, students’ reflection journals, voice and video recordings of study group activities, Facebook postings, student artifacts, focus group discussions, and individual interviews to enhance credibility (Yin, 2017). ***Observation notes*** were collected through participant observation (Kawulich, 2005), in which the researchers took the role of the observer to document participants’ interactions in the social context. The researchers also reflected on what happened during the arrangement of time and place for meeting, their facilitation of the MOOC study group (e.g., modeling, guiding focus group discussions, and providing announcements and feedback), as well as student actions and reactions toward the above-mentioned arrangements. Moreover, two trained research assistants joined the study group observation. They specifically helped document student actions and interactions, as defined as three or more participants talking to each other during their discussion (see Figure 1 for an example).

Each week, the participants were asked to submit their ***goal setting sheets***, as well as ***reflection journals*** that demonstrated their progress and thoughts on their MOOC study. The goal setting sheet assignment was designed to practice goal setting and planning, which were reported as highly related to MOOC learning performance (Pursel et al., 2016; Kizilcec et al., 2017). On the other hand, the weekly reflection journals had been useful not only to provide information about the participants’ inner states such as motivation, aspiration, and action plans, but they were helpful for the researchers to adjust plans and facilitation of group discussion every week.

As mentioned earlier, each week after the student-led discussion, the researchers facilitated a ***focus group meeting*** that captured the current status and feedback of the group members. This could help the researchers identify the participants’ instant reflections as well as their changes over time. At the end of the 6-week study group, the participants were interviewed individually for about 90 min to understand their experiences and attitudes from their personal perspectives. The interview outline included five aspects:

1. Self-evaluation of the learning process and strategies;
2. Topics/issues worked together and problems resolved;
3. Leadership experience and perceived effectiveness;
4. Group interactions and interpersonal relationships;
5. Situations and reasons to utilize face-to-face or Facebook discussions;
6. Effectiveness/barriers/suggestions about the MOOC study group.

Group meetings were video-recorded with the consent of all participants. Each session was captured by three cameras (on one notebook computer and two tablets) from different angles to record group interactions and computer operations. Recorded videos were then coded into video logs based on major events in the meetings (see Table 3 for an example). Eventually, video logs were assembled for further analysis.

**TABLE 3:** A sample video log.

Participants: Omar, Burton, Elizabeth	Date: 6/5	Time: 6 min 15 s–7 min 07 s	Source: Camera 1
**Activity**: Watching videos together		**Interaction behavior**: Participants searched for lecture videos related to Question 19 and then discussed the content while watching the videos together.	
**Main content:** Burton operated the video player, and the others moved their seats closer to watch the video together. Maggie did not participate in this part of the discussion because her seat was too far away.			

Lastly, we collected student artifacts such as study notes and PowerPoint slides shared by group members. Those artifacts were used to supplement or cross-validate results from other data sources. Furthermore, every message posted on Facebook was collected and analyzed. Together, the multiple data sources provided rich information to examine student interactions in face-to-face and online contexts, as well as participants’ perceptions of the blended mode study group to learn MOOC together.

### Data Analysis

Recorded interviews and focus group meetings were transcribed, and the verbatim was then processed with (Nvivo) 10. The interview and focus group discussion verbatim were analyzed with qualitative approaches (Patton, 2014). The researchers conducted the first cycle of coding, namely identifying the unit of analysis and segmenting the original texts accordingly. A structural coding (Saldaña, 2016) was performed according to the initial coding scheme, so each meaningful text segment was related to a specific research question. When the text did not fit the initial coding scheme, descriptive coding was then applied to the text, in turn, the whole coding scheme expanded to accommodate all text segments.

Two experienced research assistants joined the analysis in the second coding cycle, including free coding and focused coding. Inconsistencies between coders were resolved during regular research meetings led by the researchers. During the whole process, simultaneous coding was applied so that the same text could be coded under different labels and later be interpreted in multiple levels and by multiple perspectives (Miles and Huberman, 1994; Saldaña, 2016).

Video recordings were processed somewhat differently. Based on our research purpose, we used “*interaction*” as the unit of analysis, which was defined operationally as “three or more participants gather together to work on a certain issue.” The extracted interactions were further categorized in reference to the following questions:

1. In what conditions do the group members discuss together?
2. What are the contents/issues that they work together?
3. What are the main concerns/focus during that interaction?
4. What are the results of that interaction? (e.g., when resolving a difficult problem together)?
5. What are the member roles (e.g., leader, follower, help seeker, resolver, etc.) and reactions during that interaction?

As with Facebook postings, *via* content analysis (Gerbic and Stacey, 2005), each forum thread was tagged by date, week, the name of the author, the number of replied posts, total reads, total “thumb ups,” and content of posts and replies. We further used Microsoft Excel to sort those threads by a combination of tags, based on which we generated percentage tables, pie charts, and line graphs that portrayed the participants’ patterns of interactions on Facebook.

### Reliability and Validity

“Consistency” is commonly used as an indicator to evaluate data reliability in qualitative research (Merriam, 2002). In qualitative studies, the “inter-rater reliability” or the “degree of agreement” are calculated to signify the consistency of coding. A high percentage agreement between coders means that other trained researchers would be most likely to categorize the same data into the same codes following the same coding procedure. In this study, we ran a coding comparison query in Nvivo. The initial average of percentage agreements was 96.12%, indicating appropriate consistency/reliability in the field of computer-supported cooperative work (CSCW) (McDonald et al., 2019). All final coding was set in regular research meetings, wherein differences in coding were discussed and determined upon agreement of coders.

Validity in qualitative research often means the extent to which the results represent participants’ views on the events or experiences (Creswell and Miller, 2000). Qualitative researchers employ triangulation, member check, think description, etc., to establish the validity of their studies (Denzin and Lincoln, 2011). In this study, we applied investigator triangulation and data triangulation (Golafshani, 2003) as validity procedures. For investigator triangulation, two major researchers and two research assistants worked together to collect data: the two major researchers were participant-observers while the other two research assistants observed the group from pure outsiders’ perspective without involvement. This arrangement helped balance between emic and etic perspectives (Helfrich, 1999), and reduced the bias of individual researchers (Archibald, 2016). Also, the codings from different coders were cross-examined and differences in coding were solved by discussion. As with data triangulation, we collected data from multiple methods (e.g., observations, interviews, and video recordings). Data from different sources were cross-examined to find any contradictions or inconsistencies in findings (Cohen et al., 2017). Again, the disparity of data was discussed and resolved during regular research meetings.

## Results and Discussion

### RQ1: What Is the Pattern of Massive Open Online Course Study Group Interaction in Face-to-Face Meetings?

**FIGURE 2:**
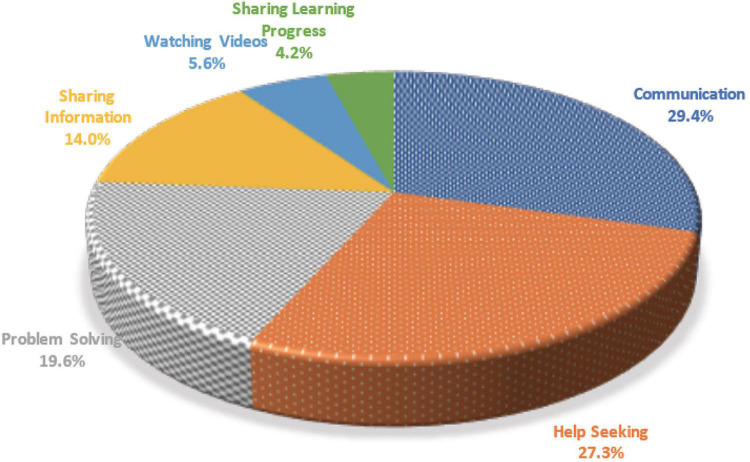
Illustration of interactions in face-to-face meetings.

A total of 143 face-to-face interactions were retrieved from the recorded videos, of which six categories of interaction were further identified (see Table 4 and Figure 2), including (1) Communication, (2) Help seeking, (3) Problem solving, (4) Sharing information, (5) Sharing learning progress, and (6) Watching videos, from the highest to the lowest frequency. ***Communication*** means formal and informal exchanges such as chatting, discussion of personal life, or arranging schedules for the study group, which may not be directly related to the learning materials.

**TABLE 4:** Categories of interaction in face-to-face meetings.

Types of activities	#	Percentage	Description
Communication	42	29.4%	Discussing personal life or arranging the schedule
Help seeking	39	27.3%	Asking questions or giving advice
Problem solving	28	19.6%	Explaining the concepts or working out problems together
Sharing information	20	14.0%	Providing information
Watching videos	8	5.6%	Watching/re-watching lecture videos
Sharing learning progress	6	4.2%	Sharing personal learning experience or current progress on particular questions

*Help Seeking* means asking questions or giving advice to other group members. A related category is *Problem Solving*, which usually happened when the participants were working together to resolve quiz problems, for example,

Omar: Did you get the right answer to question 16?
Maggie: No, I didn’t. What is the correct answer of 16?
Omar: Which one did you choose? I calculated and got the answer of 4.8, but it was wrong!
Burton: I wrote 7.3.
Elizabeth: I wrote 16 points and some more.
Omar: Ah! Then we got 13.3!
Burton: This is how it is calculated.
Omar: I was thinking that if we use 1 to divide it then it would be… just choose 13.3 first! 0.7 plus 2.9 times 0.29… so we got 0.177, it should be a 16.6 traction rate. I thought of dividing the related number, but 1 divided by 0.177 wasn’t right.
Burton: 1 divide 0.177…
Elizabeth: 8.57
Omar: No! It’s not correct! 0.7 divide 0.06
Burton: Then we got 16.7…
Omar: Yes! 16.7, 16.66 points and a little more.

(0605_193649_Samsung_11:05–14:16)

In this study, participants came from different majors and did not have much background in marketing. During discussions, they ***Shared Information*** to clarify concepts and key terms. In such open discussions, participants freely expressed their own interpretations of the concepts or shared information they collected when they studied individually. In the following excerpt, Omar contributed what he knew about Biology and applied it to the discussion of the cycle time of the product in Marketing:

(Maggie stood up and listened)
Omar: For example, 1 divided by 0.06.
Omar: Because it’s a traction rate, it means that if you want to calculate the cycle time of life, then it needs to be divided by 1, and you will get the answer of …I don’t know how to explain it.
Omar: Just like we what we learned from Biology! You have learned that before, right? We had calculated some types of the cycle time of life.
Elizabeth: Yes, but I forgot.

(0605/NB/video2_ 18:06–19:24).

Lastly, the study group members watched videos together when someone used lecture videos on Coursera as evidence to support his or her opinion, or when they want to recall the content in the videos that might help them solve problems. During ***Video Watching*** interactions, usually, a group member retrieved a lecture video and provided interpretations, and the remaining participants offered feedback or asked further questions:

Omar: Burton, play the video for everyone, please.
(Watching the video…)
Omar: I will interpret it as “do you want to upgrade your car with some accessories of sports cars?” For example, the sports car’s chair.
Burton: The accessories of a sports car?
Elizabeth: oh!
Omar: So, you mean you are not talking about the sports bag you just said.
21:22–26:30:
Omar: (watching the video) He said it just now… “those two numbers multiplied together result in one…” (feeling confused), maybe it’s just an expression.
Burton: Wait a minute… (replaying the video and listening carefully to the video) … it decreases by 0.2 each year, and in five years, nothing will be left.
Omar: At the beginning, the total number is one. And it decreases by 0.2 each year, it becomes zero at the end of the fifth year.
Elizabeth: Oh, I see.
Omar: Then we don’t need to watch the video all over again. Do the math- and you’ll figure it out..

*0605_Samsung_04:15–07:07.*

According to Pursel et al. (2016), video watching behaviors were positively related to MOOC course completion. It seems that our face-to-face meetings had created a supportive environment for watching videos together with peers that can in turn contribute to students’ completion of the MOOC course. Moreover, looking across the categories of face-to-face interactions, we found the categories were all related to solving problems in assignments and quizzes. More specifically, more than half (53%) of the face-to-face interactions were associated with assignment-related activities, including *Watching Video*, *Help Seeking*, and *Problem Solving*. The above instances showed that the group meeting conversations were contributive to their co-construction of knowledge. Also, many conversations reflected the process of peer scaffolding in order to work out the problems.

**FIGURE 3:**
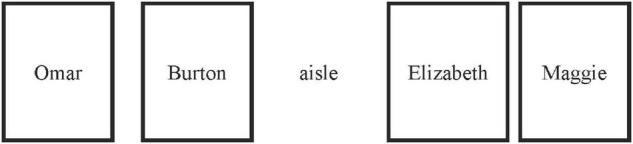
Original seat arrangements before week 3.

**FIGURE 4:**
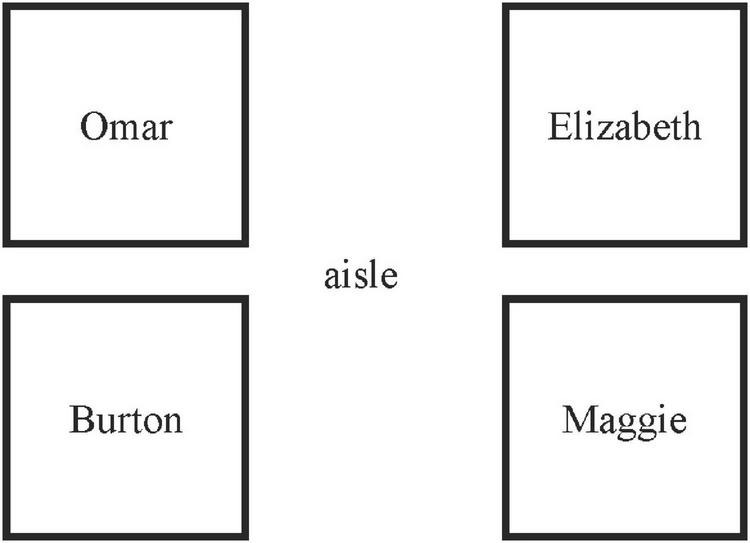
Adjusted seat arrangements in week 3.

It is also important to note that, the seats had been changed since Week 3. In Week 2 when the study group started officially, students had no way to face each other because the seats were linearly arranged. The desks were arranged in a row, all facing to the front (see Figure 3). The researchers found that Maggie only talked to Elizabeth and was easily ignored during group discussions. Therefore, in Week 3 we changed the seat arrangement as illustrated in Figure 4. It turned out that the overall interactions among group members improved significantly, and Maggie talked more and started to seek help from others.

### RQ2: What Is the Pattern of Massive Open Online Courses Study Group Interaction in Online/Facebook Postings?

At the end of the 6-week study group, a total of 32 Facebook threads (including posts and replies) were retrieved from the Facebook group. All the group members posted at least four messages on Facebook. Compared to the study of Swinnerton et al. (2017) wherein only a third of MOOC learners posted at least one comment on their course website, the online interactions in our study group seemed quite intense. It seems that compared to post messages on a larger course group, the participants may be more willing to post messages on the smaller study group which they created and experienced a sense of community. As evidenced by the study of Pursel et al. (2016), students’ engagement in online postings and comments could strongly predict their MOOC completion.

**FIGURE 5:**
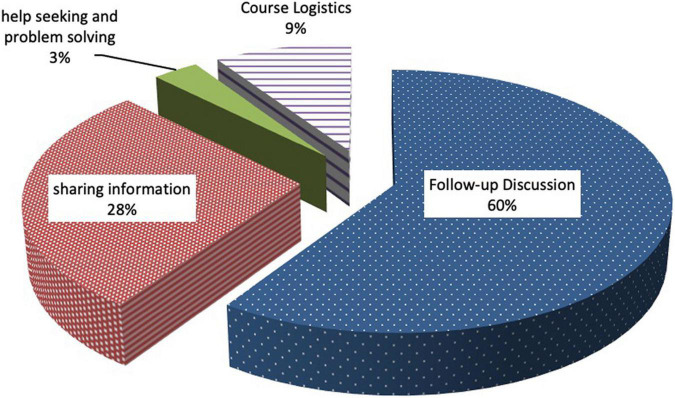
Illustration of interactions in Facebook postings.

Table 5 and Figure 5 portray the four categories of online/Facebook interactions through thematic analysis, including (1) *Follow-up Discussion*, (2) *Sharing Information*, (3) *Course Logistics*, and (4) *Help Seeking and Problem Solving*, from the highest to the lowest frequency. Among the four types of interactions, *Sharing Information* usually included artifacts or supplementary learning materials. For example, some posted vocabulary lists that had been discussed in group meetings. In addition, the participants shared websites related to the topics they discussed during face-to-face meetings. Such kinds of online sharing could be regarded as the extension of their face-to-face discussions to support or defend their previous arguments. de Lima and Zorrilla (2017) found that peer sharing of information was perceived as very informative by their MOOC students. Similarly, in the Liu et al. (2016) study, more than half of the students agreed that the Facebook group was useful for their MOOC learning, and one of the most useful aspects was resources shared with the group. Interestingly, in the Liu et al. (2016) study, resource sharing was the most frequent type of online posting while in the present study it ranked second among the four types of postings.

**TABLE 5:** Categories of interaction in Facebook postings.

Types of posting	Frequency	Description	Example
Follow-up discussion	19 (60%)	Follow-up of previous topics in group meetings	“Keywords (chapter 3, week …)”
Sharing information	9 (28%)	Repost or post course-related information	Provide hyperlink for a related article, such as “https://www.facebook.com/useMyoops”
Course logistics	3 (9%)	Discussing meeting schedules	“Does anyone want to take another Coursera course?”
Help seeking and problem solving	1 (3%)	Ask help for assignments or discussing course materials	“How do you take notes?”

*Help Seeking and Problem Solving* contained the fewest postings (3%) among the Facebook interactions. It is possible that our participants had already used the chance of face-to-face meetings to work together and help each other. Another possible reason is that, from the perspective of the Connectivist Interaction and Engagement framework (CIE, Wang Z. et al., 2014), providing help requires deeper cognitive engagement and may reduce the frequency of such postings. Follow up Discussion, which requires higher levels of cognitive engagement, contained as much as 60 percent of the Facebook postings. This could be interpreted that many discussion postings were the follow-ups of previous face-to-face discussions instead of new discussions, and to some extent, it reduces the cognitive engagement required for such postings. In the analysis of the cognitive presence of a blended mode learning community, Vaughan and Garrison (2006) reported a similar observation that cognitive discussions were less likely to be initiated in online environments. They found that the “triggering event,” which was the beginning phase of the inquiry process, was more frequent in face-to-face meetings (13%) than online forum discussion (8%).

**FIGURE 6:**
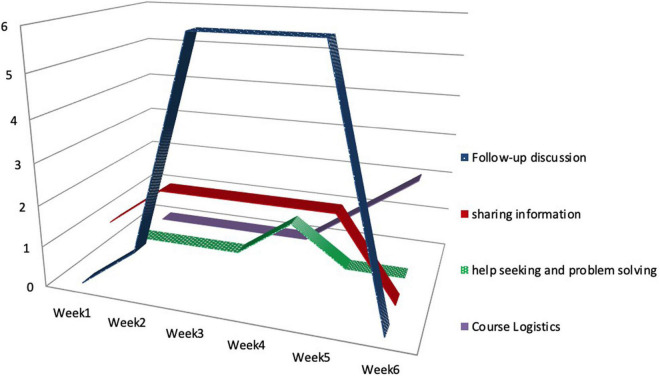
Frequencies of Facebook postings across the 6-week session.

Although not all members were keen to post their ideas on the social network, most of them followed similar patterns regarding the numbers of postings across time (see Table 6 and Figure 6 for details). Namely, they posted more articles in the middle of the course and far less at the beginning and the end. This pattern may provide evidence that the group members were using Facebook to assist their face-to-face discussions. Another possible reason may be the rearrangement of seats in week 3. The participants started to discuss in a circle, facing each other, and only went back to their computers when needed. The change of seat arrangement might explain some burst of posting as they also interacted more in person.

**TABLE 6:** The frequency of weekly postings by person.

Participants	Type of posting	Week 1	2	3	4	5	6
Maggie	Follow-up discussion	0	0	2	1	1	0
	Sharing information	1	2	2	1	1	0
	Help seeking	0	0	0	1	0	0
	Course Logistics	0	0	0	0	1	1
Omar	Follow-up discussion	0	0	2	1	0	0
	Sharing information	0	0	0	0	1	0
	Help seeking	0	0	0	0	0	0
	Course Logistics	0	0	0	0	0	0
Burton	Follow-up discussion	0	0	1	3	2	0
	Sharing information	0	0	0	1	0	0
	Help seeking	0	0	0	0	0	0
	Course logistics	0	0	0	0	0	0
Elizabeth	Follow-up discussion	0	1	1	1	3	0
	Sharing information	0	0	0	0	0	0
	Help seeking	0	0	0	0	0	0
	Course Logistics	0	0	0	0	0	1
Total		1	3	8	9	9	2

It is worth mentioning that, each member appeared to prefer different types of posting. For example, Elizabeth only posted threads of *Course Logistics* and *Follow-up Discussion*, whereas Omar and Burton contributed to *Information Sharing* and *Follow-up Discussion*. Maggie, who engaged in Facebook discussions more than anyone else, posted threads of all kinds. It seems that the differences in postings among group members reflected the different roles they played in the MOOC study group. The difference in postings also corresponds with our previous profiling of their backgrounds, characteristics, and preferences in section Participants.

### RQ3: How Do Massive Open Online Courses Study Group Members Perceive Their Experience of Interactions in the Face-to-Face and Facebook Contexts?

In this study, participants valued the opportunity to learn from each other in the study group meetings. In particular, they believed that they comprehended learning materials more easily when they watched lecture videos together during face-to-face sessions. All of them finished all required assignments and quizzes, and most of them (three out of four) earned “Statement of Accomplishment” from the course for their achievements. The participants thought that working together as a group helped them learn better. Elizabeth mentioned that, compared to listening to the instructor alone, other members could rephrase the learning materials and explain to her in ways that she could comprehend more easily. Burton elaborated this by saying “…it would be easier to understand those concepts when students who already understand it to explain to me in simpler and clearer ways.” Another member, Omar, also agreed and said, “Sometimes you cannot grab the main ideas from the video; however, when watching videos together, some group members who understand more on the topic may give extra information to relate this….then I can understand what the video is all about.”

Regarding Facebook, the participants deemed it a useful tool to share information, provide timely help, and extend face-to-face communications without the limitation of time and space. According to Burton, “Facebook is a space for follow-up discussions…and another benefit of it is that you can upload and share files and materials with others, it’s a space for sharing.” Elizabeth valued Facebook for more immediate responses to resolve problems: “… if you have some questions you can post it on Facebook, and then someone may help you resolve the problem.” Omar also said,

“…it’s a way to connect to each other because we only meet once a week. When you think of something that you forgot to share in face-to-face meetings, you can always share it on Facebook. So it is a place where you can share what you think, as well as catch up on what you forgot to share. Also, when you raise a question on Facebook, we can have extra discussions. I think it is nice that we have the Facebook group, a communication tool…because it is not very convenient to discuss over the phone. And it’s free and all of us can see it!”

During the interview, one participant, Elizabeth explained the difference between discussion on Facebook and face-to-face interactions. When she was asked to identify what she would post on Facebook and what she would prefer to discuss in person, she said, “…for trivial matters such as meeting time, and those which did not directly relate to the course content, posting on Facebook should be just fine….and the core content of the course, it would be good to discuss in person.” It is also noteworthy that Facebook facilitated prolonged communications among the study group members: after the end of the 6-week study group, the participants used Facebook to communicate with each other and even scheduled a face-to-face reunion. It appears that Facebook had played an important role in maintaining the connections among study group members; also a sense of community had been escalated with the help of the Facebook group.

In this study, we examined the interaction patterns and perceptions of students in the 6-week MOOC study group. The interactions in face-to-face meetings and Facebook showed some similar patterns, as both contexts enabled *Help Seeking*, *Problem Solving*, and *Information Sharing* as categories of interaction. Although participants initiated more new issues in the face-to-face meetings, they continued with their discussions on Facebook. It seems that deep and immediate interactions were better achieved in face-to-face meetings, whereas the Facebook group offered a platform for the situation called for asynchronous interactions. In addition, problem-solving was commonly seen in face-to-face discussions, whereas more information and resource sharing were prevalent on Facebook. Newman (1995) argued that, compared to face-to-face meetings, online asynchronous discussions were less effective for problem-solving. This may explain why our participants devoted their valuable meeting time to doing assignments; otherwise, it would be much more difficult to accomplish it *via* online discussions.

Comparing group activities on Facebook and in face-to-face meetings, *Follow-up Discussion* on Facebook was essentially the extension of previous group discussions in face-to-face meetings. On the other hand, in face-to-face meetings, there were more “working together” activities in which all members participated in the same activities together at the same time and in the same place (e.g., watching videos or working on assignments together). Working together may not necessarily or directly facilitate interactions, but it generated discourse spaces and shared experiences critical for community building and knowledge co-construction.

## Conclusion, Limitations, and Future Directions

In Moore (1989) highlighted the criticality of interaction in distance learning. It holds true even, three decades later, in the context of MOOCs. In this study, we carried out a “blended mode,” student-led MOOC study group to promote interactions and social learning. Findings indicated that, overall, the blended mode MOOC study group was helpful for promoting communication, providing help, resolving problems, and exchanging ideas and information among group members. Moreover, face-to-face meetings and online discussions both exerted their unique strengths and functions that aligned with different learning situations and learner preferences. Online study groups offered generous spaces for learners to continue their discussions initiated in their face-to-face meetings and extended their learning. As such, we would like to recommend this blended format preferably to exert the full potential of the MOOC study group. On the other hand, *self-regulated learning (SRL)* has been increasingly emphasized in higher education; for example, many universities in Taiwan are now supporting/subsidizing college students to formulate spontaneous study groups to learn something to their interest. We deem MOOCs can be an ideal target for students to learn/explore together, and our blended mode study group approach would serve as a practical, easy to implement, and effective way to promote motivation and learning of MOOCs.

This study has its limitations. First of all, this study contains only four participants, which may to some extent limit the generalizability of the study results. Despite that, from the perspective of Critical Realism, one single case may be valuable to offer unique insights (Easton, 2003), as “one talking pig is sufficient to prove that pigs can talk” (Editorial Nature Neuroscience, 2004, p.93). Research communities in Neuroscience, Management, Social Science, etc., have recognized the potential of a single case study in the research fields (Editorial Nature Neuroscience, 2004; Silverman, 2013; Mariotto et al., 2014; Ozcan et al., 2017). In any case, more studies are recommended to replicate our blended mode MOOC study group in different cultures, subject areas, age groups, etc., to establish the generalizability of the MOOC study group in a blended format. Comparisons of interaction patterns across studies are also helpful for generating insights into the design of MOOC interactions in different contexts.

Another limitation arises with the prevalence of COVID-19 when a pandemic like this has posed a threat to face-to-face encounters such as in-person study groups. In such cases, the online/Facebook portion of our MOOC study group is still useful for promoting communications and social interactions for MOOC studies. More promisingly, as COVID-19 vaccines are becoming widely available around the globe, face-to-face group discussions can be expected to resume in the foreseeable future. We believe that the blended-mode study group may further serve as a useful methodology/pedagogy to prepare for online learning in the post-COVID-19 era.

The present study contributes to our knowledge base by supporting the tenability of MOOC study groups, portraying the utility of the study group approach to support blended learning of MOOCs, and analyzing interaction patterns that look into the structure and discourse during the study group process. It is our strong belief that continued investigation and improvement of MOOC interaction design will make MOOC learning more intriguing and fulfilling, helping us become lifelong learners in the twenty-first century.

## Data Availability Statement

The datasets presented in this article are not readily available because the identities of participants may be revealed in the raw data. Requests to access the datasets should be directed to the corresponding author P-JC, pinju@mail.mcu.edu.tw.

## Ethics Statement

Ethical review and approval was not required for the study on human participants in accordance with the local legislation and institutional requirements. The patients/participants provided their written informed consent to participate in this study.

## Author Contributions

Both authors listed have made a substantial, direct, and intellectual contribution to the work, and approved it for publication.

## Conflict of Interest

The authors declare that the research was conducted in the absence of any commercial or financial relationships that could be construed as a potential conflict of interest.

## Publisher’s Note

All claims expressed in this article are solely those of the authors and do not necessarily represent those of their affiliated organizations, or those of the publisher, the editors and the reviewers. Any product that may be evaluated in this article, or claim that may be made by its manufacturer, is not guaranteed or endorsed by the publisher.
